# Clues for two-step virion infectivity factor regulation by core binding factor beta

**DOI:** 10.1099/jgv.0.000749

**Published:** 2017-05-18

**Authors:** Youwei Ai, Jianzhang Ma, Xiaojun Wang

**Affiliations:** ^1^​ College of Wildlife Resources, Northeast Forestry University, Hexing Road, Harbin 150040, PR China; ^2^​ State Key Laboratory of Veterinary Biotechnology, Harbin Veterinary Research Institute, Chinese Academy of Agricultural Sciences, Harbin, PR China; ^†^​ Present address: National Institute of Biological Sciences, Beijing, PR China.

**Keywords:** lentivirus, HIV-1, SIVmac, vif, CBF-β, APOBEC3

## Abstract

Lentiviruses threaten human and animal health. Virion infectivity factor (Vif) is essential for the infectivity of most lentiviruses, except for the equine infectious anaemia virus (EIAV). Vif promotes viral infectivity by recruiting a Cullin-based E3 ligase to induce the degradation of a class of host restriction factors, named APOBEC3. Core binding factor beta (CBF-β) is necessary for several primate lentiviral Vif functions, including HIV-1 Vif. Although much progress has been made in understanding the contribution of CBF-β to Vif function, the precise mechanism has not yet been fully elucidated. In this study, we found that an interaction with CBF-β altered the oligomerization and subcellular distribution pattern and increased the stability of two primate lentiviral Vifs, HIV-1 Vif and Macaca simian immunodeficiency virus (SIVmac) Vif. Moreover, using a CBF-β loss-of-function mutant, we demonstrated that the interaction between CBF-β and Vif was not sufficient for Vif assistance; a region including F68 in CBF-β was also required for the stability and function of Vif. For the first time, this study separates the binding and regulating processes of CBF-β when it is promoting Vif function, which further extends our understanding of the biochemical regulation of Vif by CBF-β.

## Abbreviations

BIV, bovine immunodeficiency virus; CAEV, caprine arthritis encephalitis virus; CBF-β, core binding factor beta; EIAV, equine infectious anemia virus; FIV, feline immunodeficiency virus; HIV, human immunodeficiency virus; MVV, maedi-visna virus; SIV, simian immunodeficiency virus; Vif, virion infectivity factor.

## Introduction

Common lentiviruses include human immunodeficiency virus (HIV), simian immunodeficiency virus (SIV), feline immunodeficiency virus (FIV), bovine immunodeficiency virus (BIV), caprine arthritis encephalitis virus (CAEV), maedi–visna virus (MVV) and equine infectious anaemia virus (EIAV) [1, 2]. Among these, EIAV is the only lentivirus that does not encode a virion infectivity factor (Vif) to promote viral infectivity [3, 4]. Most lentiviruses benefit from Vif protein by recruiting a Cullin–Elongin B/C–Rbx-based E3 ligase to induce cellular restriction factor APOBEC3 family member degradation [5–11], relieving the lethal G-to-A editing of APOBEC3 to a viral plus genome [6, 12, 13]. Recent studies have proposed that core binding factor beta (CBF-β) is required for the function of several primate lentiviral Vifs, including HIV-1, Macaca SIV (SIVmac) and African green monkey SIV (SIVagm), but is not required for non-primate lentiviral Vif function [1, 14–18].

CBF-β is a host protein that interacts with RUNX family proteins to activate or repress the transcription of various genes, including APOBEC3G (A3G) in certain cell lines [19]. Moreover, CBF-β can interact with HIV-1 Vif protein, increase the biosynthesis and stability of Vif, alter the structural conformation and strengthen the interaction between HIV-1 Vif and Cullin5 to induce APOBEC3 degradation and increase viral infectivity [14, 20–23]. Considering the importance of CBF-β to viral fitness, disrupting the interaction between CBF-β and HIV-1 Vif is a target for antiviral therapy.

Recent biochemical and structural studies on Vif–CBF-β interaction have broadened our view. There are many amino acids in HIV-1 Vif that contribute to CBF-β binding, including W21, W38, W5, V7, I9, W11, E88W89, G84, D104, L106, I107, F115, G126, E134, Y135 and G138 (the numbers indicate the amino acid position in the HIV-1 Vif protein) [5, 24–30]. Mutating any one of these amino acids decreases the Vif–CBF-β interaction and impedes the Vif function of inducing APOBEC3 degradation. The first 14 amino acids of HIV-1 Vif are necessary for HIV-1 Vif–CBF-β interaction [20], and the first 12 amino acids of HIV-1 Vif form a direct interaction with β-strand S3 in CBF-β [24]. Deleting the first eight amino acids in HIV-1 Vif abolishes the HIV-1 Vif–CBF-β interaction [30]. Extensive regions, including loop 3, helix 4, the C terminus and an amino acid F68 in CBF-β, were proposed to be important for HIV-1 Vif binding and function [5, 18, 24, 31]. All of these studies indicate that the binding of Vif and CBF-β is required for Vif function. Any changes that decrease the association of Vif and CBF-β hinder Vif-mediated APOBEC3 degradation.

In this study, we investigated the influence of CBF-β on two primate lentiviral Vifs, HIV-1 Vif and SIVmac Vif. Our study revealed that CBF-β binds to these two primate lentiviral Vifs, decreases their oligomerization, alters the subcellular distribution pattern and increases their cellular stability. Combined with the existing results, which have consistently concluded that CBF-β-binding-deficient Vif mutants cannot induce APOBEC3 degradation, we propose that the interaction between CBF-β and primate lentiviral Vifs is the basis of Vif function. However, the binding of CBF-β to primate lentiviral Vifs is not enough for Vif regulation; the additional conformational regulation of Vif by CBF-β through a functional domain containing F68 is required for Vif stability and function. Our study proposes a new model that aids understanding of Vif regulation by CBF-β.

## Results

### Coexpression of CBF-β altered the subcellular distribution of HIV-1 Vif and SIVmac Vif

HIV-1 Vif proteins can form puncta in cells, which has been associated with their function [32]. As CBF-β can bind to HIV-1 Vif, regulate the conformation of Vif and inhibit Vif oligomerization, it is possible that CBF-β can affect the formation of HIV-1 Vif puncta. Therefore, we analysed HIV-1 Vif and SIVmac Vif proteins in the presence or absence of CBF-β coexpression via cell imaging.

**Fig. 1.**
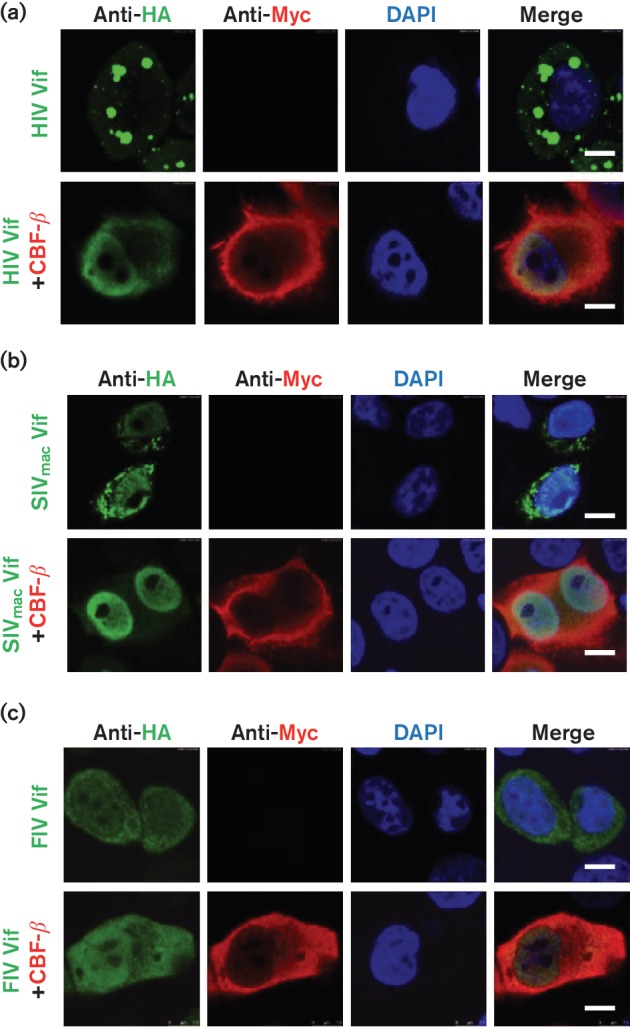
CBF-β affects the subcellular distribution of SIVmac Vif. HeLa cells were transfected with HIV Vif (a), SIVmac Vif (b), or FIV Vif (c), together with the empty vector or CBF-β expression plasmids. After 24 h, the cells were fixed, probed with antibodies against HA (Vifs) or Myc (CBF-β) and analysed by confocal microscopy. The nuclei were stained with DAPI. The scale bars represent 7.5 µm.

When C terminal double-HA-tagged HIV-1 Vif was overexpressed in HeLa cells, obvious puncta formed in the cytoplasm (Fig. 1a). However, the coexpression of CBF-β abolished the puncta of HIV-1 Vif (Fig. 1a). The same situation applied to SIVmac Vif (Fig. 1b). FIV Vif could not interact with CBF-β, and thus FIV Vif could be used as a negative control in this assay. However, FIV Vif did not form dots in cells, and the coexpression of CBF-β did not affect the cellular distribution of FIV Vif (Fig. 1c), excluding the possibility that the overexpression of CBF-β nonspecifically affects the localization of cellular proteins. Our results demonstrated that CBF-β altered the cellular distribution pattern of HIV-1 Vif and SIVmac Vif.

To further confirm the results found in HeLa cells, we performed the same immunofluorescence experiments in 293T cells. HIV-1 Vif formed puncta in the cytoplasm of 293T cells, and the coexpression of CBF-β decreased the puncta in the cells (Fig. S1a, available in the online Supplementary Material). Similarly, FIV Vif did not form bright dots in 293T cells, and CBF-β did not affect the cellular localization of FIV Vif (Fig. S1b).

Fixation of cells with paraformaldehyde might affect the localization of proteins in cells. To exclude the possible influence of reagents or operational progress in indirect immunofluorescence on protein localization, we generated HIV-1/FIV Vif constructs with green fluorescent protein (GFP) fused at the Vif C terminus and a construct with red fluorescent protein (RFP) fused at the C terminus of CBF-β. We transfected HIV-1 Vif or FIV Vif with an RFP control vector or a CBF–RFP vector in 293T cells or HeLa cells, respectively. We consistently observed puncta in the HIV-1 Vif-transfected cells (Fig. S2a, b); RFP on its own could not affect the distribution of HIV-1 Vif–GFP, but CBF–RFP decreased the puncta formed by HIV-1Vif–GFP in both cell lines (Fig. S2a, b). Similarly, FIV Vif–GFP was distributed evenly in the cells, and coexpression of CBF–RFP did not affect its localization (Fig. S2c, d).

HIV-1 Vif induces APOBEC3 degradation in 293T cells without additional CBF-β overexpression, and knockdown of endogenous CBF-β expression impairs HIV-1 Vif-mediated APOBEC3 degradation [16, 18]. Previously, we established a CBF-β stable knockdown 293T cell line (293T-CBF-KD) and determined that HIV-1 Vif’s capacity to degrade A3G was impaired [1]. Therefore, we checked the formation of HIV-1 Vif puncta in 293T and 293T-CBF-KD cells by both immunofluorescence and live cell imaging. We found that HIV-1 Vif puncta formed in the 293T-CBF-KD cell line; their size seemed to be same as in 293T cells (Fig. S3a). Moreover, when a GFP-tagged HIV Vif construct was transfected into cells, puncta formed in both 293T and 293T-CBF-KD cells without any obvious differences (Fig. S3b). Therefore, the knockdown of endogenous CBF-β expression did not dramatically alter puncta formation when HIV Vif was overexpressed. The reason for this finding might be that the knockdown efficiency of CBF-β was not sufficiently high [1] and/or that the level of endogenous CBF-β was not sufficient to affect puncta formation for the highly expressed HIV-1 Vif protein.

### Coexpression of CBF-β decreased the oligomerization and increased the stability of SIVmac Vif protein

Oligomerization of HIV-1 Vif protein occurred when it was expressed in eukaryotic cells or in *Escherichia coli* [29]. Coexpression of CBF-β inhibited HIV-1 Vif oligomerization to form a well-folded structure both *in vivo* and *in vitro* [24, 29]. Previously, by employing the sucrose gradient centrifugation method, we demonstrated that overexpression of CBF-β decreased the oligomeric level of HIV-1 Vif but did not affect the oligomeric status of FIV Vif [1]. CBF-β is also crucial for SIVmac function. Using the same method, we investigated the influence of CBF-β on the oligomerization of SIVmac Vif. Therefore, we transfected SIVmac Vif vectors with an empty vector or a CBF-β expression vector in 293T cells, respectively, to analyse the effect of CBF-β on Vif oligomerization.

**Fig. 2.**
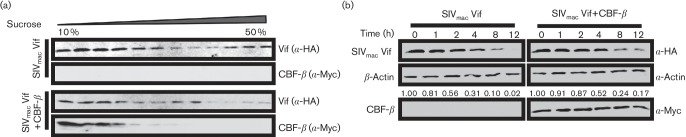
CBF-β decreases the oligomerization and increases the stability of SIVmac Vif. (a) SIVmac Vif was expressed in 293T cells with an empty vector or CBF-β, respectively. Two days later, the samples were collected, and the cell lysates were independently subjected to velocity sedimentation through sucrose gradients (10 to 50 %). Vifs were detected by anti-HA antibodies, and CBF-β was detected by anti-Myc antibodies. (b) 293T cells were transfected with SIVmac Vif and VR-CBF-mf or a control vector. After 24 h, 100 µg ml^−1^ CHX was added to the medium, and the samples were harvested at the indicated times. Vifs were detected by anti-HA antibody, CBF-β was detected by anti-Myc antibody, and β-actin was used as a loading control. The intensities of β-actin and Vif proteins in the presence or absence of CBF-β at different times were determined using the Odyssey system (Li-Cor). The numbers represent the ratio of Vifs to β-actin. These are representative data for several repeated trials.

As previously described [1] (data not shown), HIV-1 Vif was distributed in both low- and high-density sucrose cushions, and the overexpression of CBF-β decreased the oligomeric HIV-1 Vif protein. Similarly, SIVmac Vif was presented in low- and high-density sucrose cushions when cotransfected with an empty vector (Fig. 2a). Coexpression of CBF-β decreased the level of high-molecular-mass SIVmac Vif proteins (Fig. 2a), which also occurred for HIV-1 Vif, indicating that CBF-β bound to SIVmac Vif (Fig. 4b) and decreased the oligomerization of SIVmac Vifs. We also analysed the oligomerization of HIV Vif in 293T and 293T-CBF-KD cells. Similar to the results for puncta formation described above, knockdown of endogenous CBF-β expression did not dramatically alter Vif oligomerization (Fig. S3c).

Previous studies indicated that CBF-β interacts with HIV-1 Vif, thereby decreasing the proteasomal degradation of HIV-1 Vif and increasing the Vif half-life [1, 21]. We also showed that CBF-β promotes the stability of HIV-1 Vif, but not FIV Vif, via the cycloheximide (CHX) stability assay [1]. Using the same method, we cotransfected HIV 1 or SIVmac Vif vectors with an empty vector or a CBF-β expression vector in 293T cells, respectively. Then, the cells were treated with CHX to block protein synthesis, and thus we were able to observe the degradation process of Vif proteins.

As previously described, HIV-1 Vif degraded quickly when overexpressed in cells [1, 21, 23], and the coexpression of CBF-β increased the half-life of HIV-1 Vif (data not shown). Similarly, the half-life of SIVmac Vif was short when it was overexpressed (Fig. 2b). Indeed, the coexpression of CBF-β with SIVmac Vif increased the stability of SIVmac Vif in cells, although the increase was not as dramatic as that of HIV-1 Vif (Fig. 2b). Therefore, CBF-β increased the protein stability of HIV-1 and SIVmac Vifs.

### The interaction of CBF-β with HIV-1 Vif was required for Vif function

**Fig. 3.**
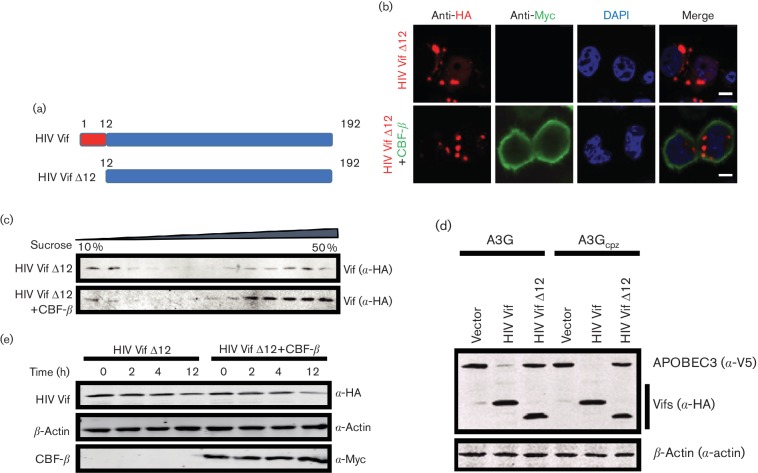
The N-terminal 12 aa of HIV-1 Vif is crucial for Vif function. (a) Schematic of the N-terminal 12 aa (red) in HIV-1. The remaining portion of HIV-1 Vif is exhibited in blue and labelled HIV Vif Δ12. Numbers indicate the amino acid position in the HIV-1 Vif protein. (b) HIV Vif Δ12 was cotransfected with the control vector or CBF-β expression vectors in HeLa cells for 24 h, and then the cells were fixed and stained with anti-HA (Vif) or Myc (CBF-β) antibodies. The scale bars represent 7.5 µm. (c) HIV Vif Δ12 was cotransfected with the control vector or CBF-β expression vectors in 293T cells, and the lysed cells were subjected to velocity sedimentation through sucrose gradients (10 to 50 %). The samples were collected and detected by Western blotting with an anti-HA antibody to detect Vif proteins. (d) 293T cells were cotransfected with HIV Vif Δ12 and the control vector or CBF-β expression vectors for 24 h. Then, 100 µg ml^−1^ CHX was added to the medium, and the samples were harvested at the indicated times. (e) HIV Vif Δ12 vectors were cotransfected with A3G or A3Gcpz into 293T cells for 24 h. The cells were collected for Western blotting with a mixture of anti-HA and anti-V5 antibodies to detect Vifs or APOBEC3s. HIV-Vif was used as a positive control.

CBF-β was able to interact with HIV-1 Vif and SIVmac Vif, and their interaction decreased the oligomerization, altered the subcellular distribution pattern and increased the stability of HIV-1 and SIVmac Vif proteins. The existing biochemical and structural studies revealed that the first 12 amino acids in HIV-1 Vif were essential for HIV-1 Vif–CBF-β interaction [21, 24, 30]. Therefore, we deleted this motif (Fig. 3a) to determine whether the HIV-1 Vif mutant (HIV Vif Δ12) was still responsive to coexpressed CBF-β when analysed by the above-mentioned biochemical assays.

We transfected the HIV Vif Δ12 vector with the empty vector or CBF-β expression vector in HeLa cells, respectively, and performed the immunofluorescence assay as described above. In contrast to the situation in wild-type HIV-1 Vif, coexpressed CBF-β could not decrease the puncta formed by the aggregated HIV Vif Δ12 proteins (Fig. 3b). In addition, HIV Vif Δ12 was mainly distributed in the high-molecular-mass form, and coexpression of CBF-β did not affect the oligomerization of HIV Vif Δ12 protein (Fig. 3c). Moreover, the overexpression of CBF-β could not affect the protein stability of HIV Vif Δ12 in the cells (Fig. 3d). This is the same situation as the CBF-β-binding-deficient HIV-1 Vif W21S or W38S mutants (data not shown). Moreover, HIV Vif Δ12 lost the ability to induce the degradation of APOBEC3 (Fig. 3e). Therefore, we concluded that the interaction between CBF-β and Vif was the prerequisite for Vif function.

### The interaction between CBF-β and Vif was not sufficient for Vif function

**Fig. 4.**
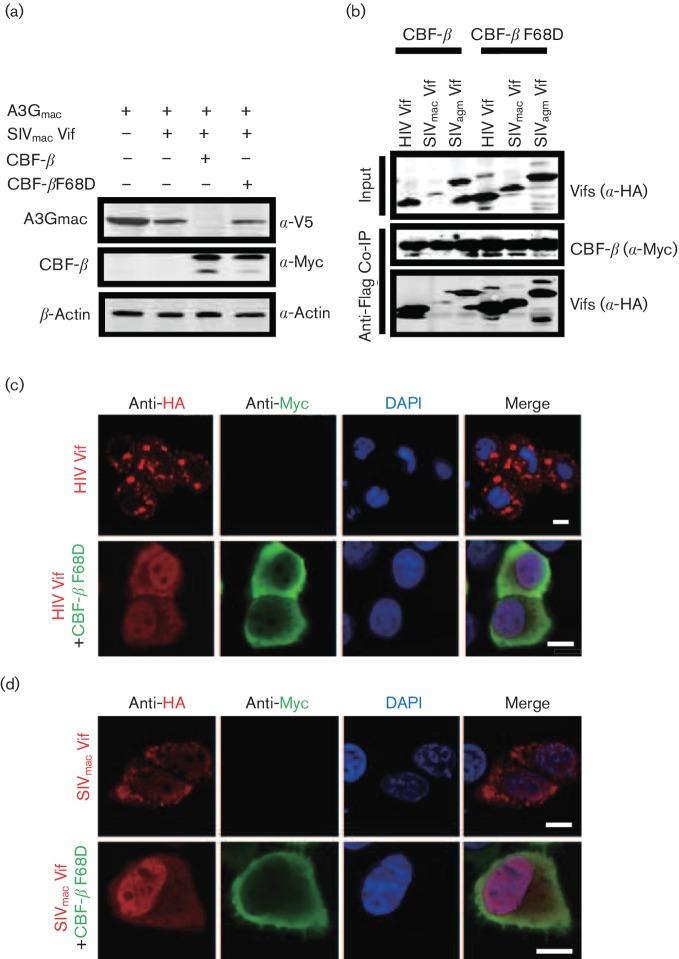

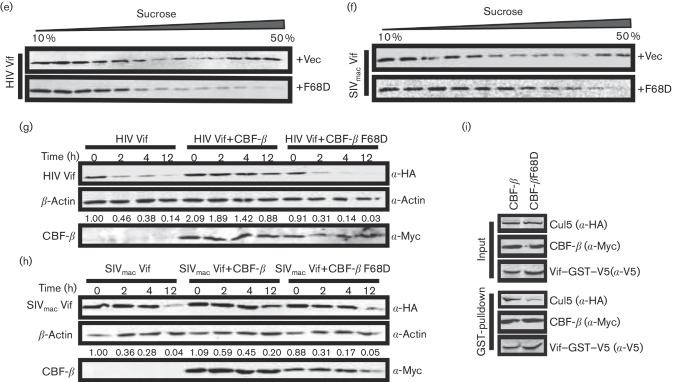
Binding of CBF-β to Vif is not sufficient for Vif regulation. (a) A3Gmac and SIVmac Vif were cotransfected with wild-type CBF-β or F68D mutant CBF-β expression vectors in CBF-β knockdown 293T cells. The lysed cells were subjected to Western blotting with an anti-V5 antibody (A3Gmac), anti-Myc antibody (CBF-β) or anti-β actin antibody. (b) Primate lentiviral Vif expression vectors were cotransfected with wild-type CBF-β or F68D mutant CBF-β expression vectors in cells for 48 h. To achieve a similar expression level, two-fold quantities of Vif plasmids were used in the F68D mutant CBF-β expression vector group. The lysed cells were subjected to anti-FLAG coimmunoprecipitation, and CBF-β or Vifs were detected by anti-FLAG or HA antibodies. F68D mutant CBF-β expression vectors were cotransfected with the control vector or CBF-β expression vectors in HeLa cells for 24 h, and then the cells were fixed and stained by anti-HA (Vif) or Myc (CBF-β) antibodies. The CBF-β F68D mutant expression vector or vector control were cotransfected with HIV Vif (c) or SIVmac Vif (d) in HeLa cells and then subjected to indirect immunofluorescence by the anti-Myc antibody (CBF-β) or anti-HA antibody (Vifs). Cell nuclei were stained by DAPI. The scale bars represent 7.5 µm. The CBF-β F68D mutant expression vector or vector control were cotransfected with HIV Vif (e) or SIVmac Vif (f) in 293T cells for 48 h, and then the lysed cells were subjected to velocity sedimentation through 10 to 50 % sucrose gradients. The samples were collected for Western blotting with the anti-HA antibody (Vifs). Wild-type or F68D mutant CBF-β expression vectors were coexpressed with HIV-1 Vif (g) or SIVmac Vif (h) in 293T cells for 24 h, and then CHX was added to the cells for the indicated times. Proteins were detected by the anti-Myc antibody (CBF-β) or anti-HA antibody (Vifs). β-actin was used as a loading control. The numbers represent the ratio of Vifs to β-actin. (i) GST-V5-tagged HIV Vif and HA-tagged Cullin5 constructs were cotransfected with CBF-β or the CBF-β F68D mutant in 293T cells. Cell lysates were subjected to GST pulldown. The proteins were detected using anti-V5 (HIV Vif), anti-Myc (CBF-β) or anti-HA antibodies (Cullin5).

First, we intended to further confirm that the CBF-β-dependent biochemical changes of primate lentiviral Vifs were correlated with the assistance of CBF-β to Vif function by introducing an amino acid mutation, F68D, in CBF-β. The F68D mutation was proposed to decrease the CBF-β–HIV-1 Vif interaction and eliminate its ability to support Vif [31]. In agreement with two independent studies [5, 31], the CBF-β F68D mutant lost the ability to promote HIV-1 Vif-mediated A3G degradation in CBF-β knockdown 293T cells (data not shown). Similarly, CBF-β F68D lost the ability to support SIVmac Vif-mediated A3Gmac degradation in a CBF-β knockdown stable cell line (Fig. 4a), indicating that F68 in CBF-β was crucial for supporting Vif function. However, when we performed the coimmunoprecipitation assay to examine the interaction between CBF-β and HIV-1/SIVmac Vif, we found that the interaction between Vif and CBF-β was not affected (Fig. 4b). We could repeatedly detect the interaction of double-HA-tagged Vif with FLAG-Myc-tagged CBF-β F68D. Moreover, primate lentiviral Vifs, but not FIV Vif, could be precipitated by CBF-β in the same system (Fig. S4) [1, 33], excluding the possible interaction between double-HA tags of Vif with CBF-FLAG affinity beads.

To further confirm the interaction of CBF-β F68D with HIV-1/SIVmac Vif, we performed the above-mentioned biochemical assays. Similar to wild-type CBF-β, CBF-β F68D decreased the aggregated HIV-1 Vif (Fig. 4c) and SIVmac Vif (Fig. 4d) puncta in HeLa cells. Moreover, coexpression of the CBF-β F68D vector, but not the control vector, decreased the oligomerization of HIV-1 Vif (Fig. 4e) and SIVmac Vif (Fig. 4f), further confirming the interaction of CBF-β F68D with HIV-1/SIVmac Vif. Our results were also consistent with a study that proposed deleting a motif including amino acid F68 in HIV Vif, which did not affect the CBF-β–HIV-1 Vif interaction [18].

However, the regulation of HIV-1/SIVmac Vif by CBF-β F68D was indeed lost, based on our studies and investigations by other groups. Thus, there must be a discrepancy between wild-type and CBF-β F68D in the regulation of Vif. Therefore, we turned to examine the change in protein stability when CBF-β F68D is coexpressed with HIV-1/SIVmac Vif. Surprisingly, when the empty vector or the wild-type or mutant CBF-β vector was coexpressed with the HIV-1 Vif expression vector in cells and subjected to the CHX stability assay, CBF-β increased the stability of HIV-1 Vif, as mentioned above, but CBF-β F68D lost the function to increase HIV-1 Vif stability (Fig. 4g). Similarly, CBF-β F68D could no longer increase the protein stability of SIV mac Vif (Fig. 4h). Furthermore, CBF-β promoted Vif–Cullin5 interaction. The CBF-β F68D mutant became less efficient than wild-type CBF-β in promoting the HIV Vif–Cullin5 interaction (Fig. 4i). As other researchers proposed that the promotion of Vif stability and Vif–Cullin5 interaction by CBF-β enhanced Vif function, our results indicated that F68 in CBF-β was important for promoting Vif function, independent of Vif binding.

## Discussion

The identification of the APOBEC3 protein as restricting the infectivity of Vif deficient HIV-1 but not wild-type HIV-1 elucidated the main function of the HIV-1 Vif protein [34]. APOBEC3, especially A3G, can block the replication of HIV-1 in many ways, e.g. by inducing the lethal editing of the viral genome [12]. The discovery of the recruitment of a Cullin5–Elongin B/C-based E3 ubiquitination ligase complex by HIV-1 Vif to induce A3G degradation explained the mechanism of Vif function in promoting viral infectivity and providing pharmacological targets for anti-HIV-1 therapy [8]. However, despite the existence of multiple studies aimed at understanding the interacting surface of E3–HIV-1 Vif–A3G, obtaining more-detailed structural information is still hampered by the poor solubility of HIV-1 Vif protein when expressed in *E. coli*. The discovery of the HIV-1 Vif chaperone protein CBF-β has conquered the obstacles existing in this field [16, 18] and has made the crystallization of HIV-1 Vif possible [29]. The overall structure of Cullin5–Elongin B/C–HIV-1 Vif–CBF-β will contribute significantly to success in the battle against HIV-1 [24, 35].

CBF-β is endogenously expressed in 293T and HeLa cells. In control 293T cells, overexpressed Vif proteins induced coexpressed APOBEC3 degradation. Knockdown of the expression of CBF-β blocked primate lentiviral Vif-mediated APOBEC3 degradation, indicating that endogenous CBF-β is essential for Vif function. However, when CBF-β was overexpressed in 293T cells, HIV-1 Vif-mediated A3G degradation increased, indicating that the level of endogenous CBF-β was not sufficient to support the function of all the overexpressed Vif [18]. Therefore, when CBF-β was coexpressed with primate lentiviral Vifs in CBF-β-expressing cells, the biochemical changes in the primate lentiviral Vifs were still obvious compared with those of the empty vector group (Figs 1 and 2).

HIV Vif easily forms high-molecular-mass multimers both *in vitro* and *in vivo*, and this characteristic is important for the function of Vif and for viral infectivity [32]. However, our results, and those from other reports [29, 36], suggest that overexpression of CBF-β could decrease oligomeric HIV Vif. Moreover, when HIV Vif binds to the Cullin5–Elongin B/C–CBF-β complex to induce APOBEC3 polyubiquitination and degradation, the components are present in a 1 : 1 : 1 : 1 : 1 ratio of monomeric complexes [24, 29, 36]. Therefore, this inhibition of oligomerization likely benefits HIV Vif in its interaction with the E3 ligase complex. Because of its inhibition of oligomerization, CBF-β may thus help HIV Vif to increase viral infectivity. However, whether the de-oligomerization of HIV Vif by CBF-β may damage other aspects of the function of HIV Vif requires further investigation.

HIV Vif is ubiquitinated and degraded rapidly in cells. A previous study proposed that an E3 ligase, MDM2, induced HIV Vif polyubiquitination and degradation [37]. Another paper published by the same group further demonstrated that CBF-β protected HIV Vif from MDM2-mediated degradation [38]. Furthermore, some research proposed that Vif is polyubiquitinated by some other E3 ligase, like Cullin5-based E3 ligase [39, 40]. CBF-β, but not a CBF-β F68D mutant, can increase Vif stability. Therefore, it is possible that CBF-β F68D lost its capacity to protect MDM2- or some other potential E3 ligase-induced degradation.

Previous studies have proposed that the mutation of F68D in CBF-β eliminates HIV-1 Vif-mediated A3G degradation, and we reached the same conclusion for both HIV-1 Vif (data not shown) and SIVmac Vif (Fig. 4a). Hultquist *et al.* explained that the defect of CBF-β in the regulation of HIV-1 Vif function was caused by the lower affinity of CBF-β F68D to HIV-1 Vif compared to wild-type CBF-β [31]. Previously, we also detected a decreased amount of precipitated primate lentiviral Vif proteins in the coimmunoprecipitation assay, which was because of the expression level of Vifs when coexpressed with CBF-β F68D was lower than when they were coexpressed with wild-type CBF-β (data not shown and Fig. 4g, h). Therefore, we increased the amount of transfected primate lentiviral Vif plasmids to compensate (the same strategy is used in Fig. 4i). We found that the interaction between primate lentiviral Vifs and CBF-β or CBF-β F68D was comparable (Fig. 4b). Moreover, coexpressed CBF-β F68D affects the oligomerization and subcellular localization of HIV-1 Vif and SIVmac Vif, similar to the situation of wild-type CBF-β (Figs 1, 2 and 4), further confirming the interaction between CBF-β F68D and HIV-1/SIVmac Vif. The lower expression level of CBF-β F68D in cells might also have accounted for the lower precipitation of HIV-1 Vif compared to that seen for the wild-type CBF-β group in Hultquist’s research [31].

Therefore, we found that a CBF-β loss-of-function mutant (CBF-β F68D) retained the interaction with primate lentiviral Vifs. The interaction between CBF-β F68D and HIV-1 Vif inhibited the oligomerization and changed the cellular localization of Vif, but did not increase its stability, promote a Vif–Cullin5 interaction or support Vif function. Our results indicated that F68D was involved in the regulation of Vif by CBF-β after the binding of CBF-β with Vif to promote the protein stability and function of HIV-1 Vif. Therefore, we separated the binding and regulating processes for CBF-β and HIV-1 Vif, which broadens our understanding of the biochemical mechanism of CBF-β action to promote HIV-1 Vif function.

A recent study proposed that the interaction of CBF-β with RUNX family proteins activates the transcription and expression of A3G in several cells [19]. HIV-1 Vif competes for CBF-β, causing decreased A3G expression. Therefore, disrupting the interaction of CBF-β with Vif would damage viral infectivity by two means, increasing A3G expression and decreasing A3G degradation. As amino acids or motifs such as the N-terminal 12 amino acids in HIV-1 Vif or F68 in CBF-β were crucial for Vif regulation, inhibitors disturbing those areas might lead to the development of new anti-HIV-1 drugs.

## Methods

### Cell lines and plasmids

HeLa and 293T cells were maintained in Dulbecco’s modified Eagle’s medium (DMEM) containing 10 % foetal bovine serum and antibiotics using standard culturing conditions. CBF-β knockdown 293T cells were obtained as previously described [1]. Plasmid expression constructs consisting of three primate lentivirus Vifs (HIV-1 Vif, SIVmac Vif and SIVagm Vif) and FIV Vif have been described previously. These Vif expression vectors were constructed in pCDNA 3.1(+) with a double-HA tag fused at the C terminus. HIV Vif Δ12 was constructed based on the HIV Vif vector by PCR technology. The CBF-β expression vector, VR-CBF-mf, has also been described previously [1]. The CBF-β F68D mutant was constructed by a site-directed point mutation method. A3G, A3Gmac, A3Gcpz were previously described [1]. HIV Vif–GFP or FIV Vif–GFP vectors were constructed by inserting Vif genes into the peGFP N1 vector. CBF-β–RFP was constructed by inserting the CBF-β gene into the pmCherry N1 vector. The HIV Vif–GST–V5 vector was constructed by fusion PCR, and the gene fragment was inserted in pCDNA 3.1(+). The VR–Cullin5–HA vector was a kind gift from Yonghui Zheng. The vectors were verified by sequencing.

### Confocal microscopy

Cells transfected with various expression vectors were fixed in 4 % paraformaldehyde in phosphate-buffered saline (PBS) and permeabilized with 0.1 % Triton X-100 24 h after transfection. After blocking with 5 % fat-free milk in PBS, the permeabilized cells were incubated with the appropriate primary antibodies in a blocking solution for 1 h at room temperature. The washed cells were then incubated with fluorescence-conjugated secondary antibodies for an additional 1 h, and the nuclei were counterstained with 4.6-diamidino-2-phenylindole (DAPI; 10 µg ml^−1^). Images were captured at ×20 magnification by a Leica digital camera attached to a fluorescence microscope (Leica DMI4000B, Germany). For live cell imaging, the cells were analysed directly under the microscope after 24 h of transfection.

### Sucrose density gradient centrifugation

Vif vectors were expressed in 293T cells expressing or not expressing CBF-β. The cells were then lysed in a buffer containing 50 mM NaCl, 5 mM Na_2_EDTA, 150 mM Tris·HCl (pH 7.6) and 1 % Triton X-100. After centrifuging at 12 000***g*** for 10 min, a 10–20−30–40–50 % sucrose step gradient was applied to the cytosolic fraction, and the gradient was centrifuged for 60 min at 163 000***g***. The proteins were separated on SDS-PAGE gels and submitted to Western blotting, as previously described [1].

### CHX Vif stability assay

Different Vif expression vectors were cotransfected with VR-CBF-mf or a vector control into cells plated in a 12-well culture plate. Twenty-four hours after transfection, the cells were treated with CHX (Sigma-Aldrich) (100 µg ml^−1^) for different times, and the cells were lysed and submitted to Western blotting, as previously described [1].

### Coimmunoprecipitation or GST pulldown

Cells were transfected with different Vif expression vectors plus a FLAG–Myc-tagged CBF-β expression vector for 24 to 48 h. Then, the cells were lysed and centrifuged to discard the membrane fractions. The supernatants were incubated with anti-FLAG M2 beads (Sigma-Aldrich) overnight, and the unbound proteins were washed five times. The bound proteins were detected by Western blotting, as previously described [1].

GST-V5-tagged HIV Vif and HA-tagged Cullin5 constructs were contransfected with FLAG–Myc-tagged CBF-β or the CBF-β F68D mutant. Thirty-six hours later, the cells were lysed. A portion of the cell lysates was evaluated by Western blotting to verify protein expression. The remaining cell lysates were incubated with 30 µl of glutathione–Sepharose beads (GE). The beads were then washed five times with lysis buffer, and the bound protein was verified by Western blotting.

### Transfections and antibodies

Plasmids were transfected into HeLa cells using Lipofectamine 2000 (Invitrogen) and into 293T cells using the standard calcium phosphate method. The antibodies used in this study included anti-Myc (Sigma-Aldrich), anti-FLAG (Sigma-Aldrich), mouse anti-V5 (Sigma-Aldrich), mouse anti-HA (Sigma-Aldrich), rabbit anti-HA (Sigma-Aldrich), anti-β actin (Sigma-Aldrich), Alexa Fluor 800-labeled goat anti-mouse IgG or Alexa Fluor 700-labelled anti-rabbit secondary (KPL), tetramethyl rhodamine isocyanate (TRITC)-anti-rabbit/mouse IgG (Sigma-Aldrich) and fluorescein isothiocyanate (FITC)-anti-rabbit/mouse IgG (Sigma-Aldrich).

## Supplementary Data

911 Supplementary File 1
