# Supplementary material for: Clues for two-step virion infectivity factor regulation by core binding factor beta
Source: J Gen Virol. 2017 May 18;98(5):1113–21. doi: 10.1099/jgv.0.000749 (PMC5656798; doi:10.1099/jgv.0.000749)
Supplement: Supplementary File 1 [file jgv-98-1113-s001.pdf]

Fig.S1

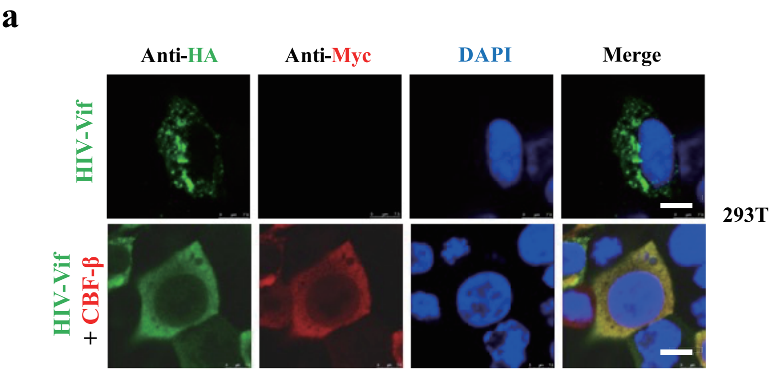
a
Anti-HA
Anti-Myc
DAPI
Merge
HIV-Vif
HIV-Vif + CBF-β
293T

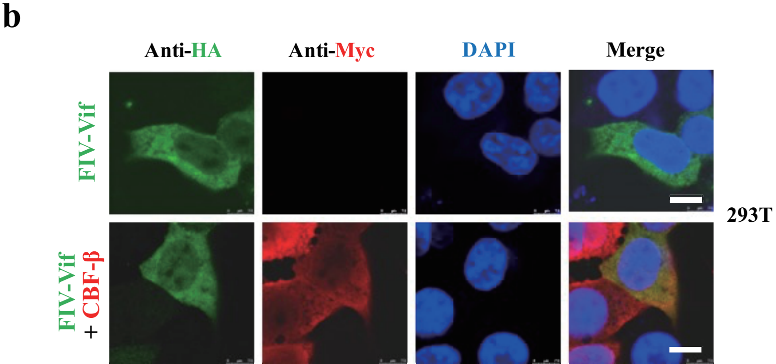
b
Anti-HA
Anti-Myc
DAPI
Merge
FIV-Vif
FIV-Vif + CBF-β
293T

Fig.S2

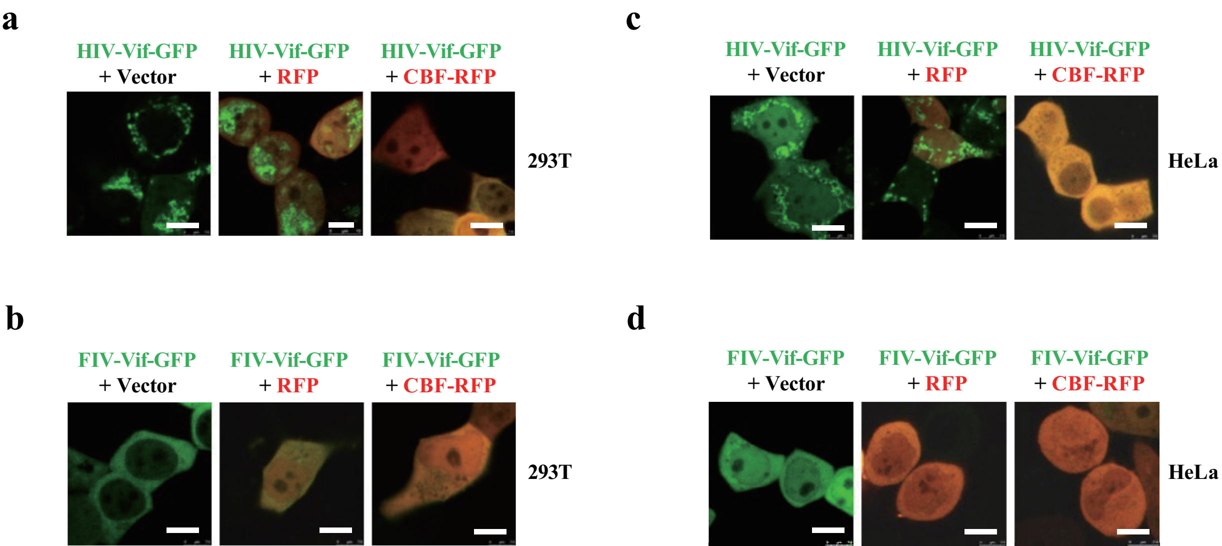
a
HIV-Vif-GFP + Vector
HIV-Vif-GFP + RFP
HIV-Vif-GFP + CBF-RFP
293T
b
FIV-Vif-GFP + Vector
FIV-Vif-GFP + RFP
FIV-Vif-GFP + CBF-RFP
293T
c
HIV-Vif-GFP + Vector
HIV-Vif-GFP + RFP
HIV-Vif-GFP + CBF-RFP
HeLa
d
FIV-Vif-GFP + Vector
FIV-Vif-GFP + RFP
FIV-Vif-GFP + CBF-RFP
HeLa

Fig.S3

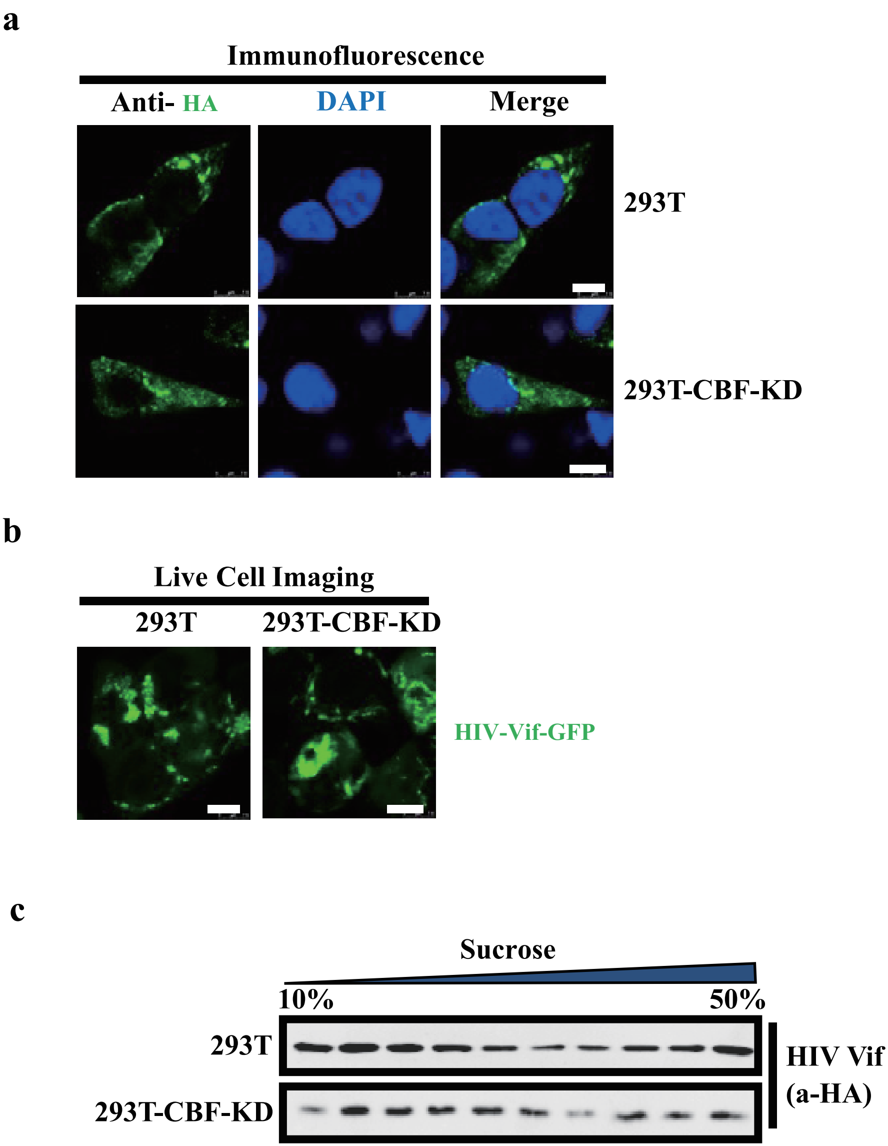
a
Immunofluorescence
Anti- HA
DAPI
Merge
293T
293T-CBF-KD
b
Live Cell Imaging
293T
293T-CBF-KD
HIV-Vif-GFP
c
Sucrose
10%
50%
293T
293T-CBF-KD
HIV Vif
(a-HA)

Fig.S4

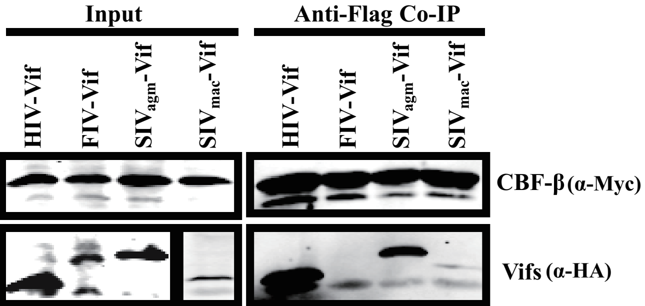

Input
Anti-Flag Co-IP
HIV-Vif
FIV-Vif
SIV_{agm}-Vif
SIV_{mac}-Vif
HIV-Vif
FIV-Vif
SIV_{agm}-Vif
SIV_{mac}-Vif
CBF-β (α-Myc)
Vifs (α-HA)

FIG S1: CBF-β changes the subcellular distribution of HIV-1 Vif in 293T cells. The CBF-β expression vector or empty vector were cotransfected with HIV-Vif (a) or FIV-Vif (b) in 293T cells for 24 hours, and then the cells were fixed and stained with the anti-Myc antibody (CBF-β) or anti-HA antibody (Vifs). DAPI was used for staining the nucleus. The scale bars represent 7.5 μm.

FIG S2: CBF-β decreases the puncta of HIV-1 Vif in live cell imaging. GFP tagged HIV-Vif (a) or FIV-Vif (b) expression vectors were coexpressed with the empty vector or the RFP or CBF-β-RFP expressing constructs in 293T cells. The protein expression in the live cells were analyzed under confocal microscopy. Similarly, GFP tagged HIV-Vif (c) or FIV-Vif (d) expression vectors were coexpressed with the empty vector or the RFP or CBF-β-RFP expressing constructs in HeLa cells, and the protein expression and localization were examined by confocal microscopy. The scale bars represent 7.5 μm.

FIG S3: Endogenous CBF-β does not dramatically affect the puncta formation and oligomerization of HIV-1 Vif in 293T cells. (a) HA-tagged HIV-Vif expression vectors were transfected into 293T or 293T-CBF-KD cells for 24 hours, and then the cells were fixed and stained with anti-HA antibody. DAPI was used for staining the nucleus. The scale bars represent 7.5 μm. (b) GFP-tagged HIV-Vif expression vectors were expressed in 293T or 293T-CBF-KD cells. The HIV-Vif protein localization in the live cells were analyzed via confocal microscopy. The scale bars represent 7.5 μm. (c)

HA-tagged HIV-Vif expression vectors were transfected into 293T or 293T-CBF-KD cells. Two days later, the transfected cells were collected, and the cell lysates were independently subjected to velocity sedimentation through sucrose gradients (10% to 50%). HIV-Vif proteins were detected with anti-HA antibodies.

FIG S4: Specificity of the interaction between primate lentiviral Vifs and CBF-β. HA-tagged Vif expression vectors were cotransfected with Flag-Myc-tagged CBF-β expression vectors in 293T cells for 48 hours. The lysed cells were subjected to anti-Flag Coimmunoprecipitation, and the proteins were detected by anti-Myc antibody (CBF-β) or anti-HA antibody (Vifs).
